# A Novel Hemizygous CDX4 Missense Variant Identified in a Taiwanese Man with Severe Teratozoospermia

**DOI:** 10.3390/medicina62081508

**Published:** 2026-08-05

**Authors:** Chin-Fong Au, Ya-Yun Wang, Tsung-Hsuan Lai, Chying-Chyuan Chan, Chih-Chun Ke, Shiu-Dong Chung, Ying-Hung Lin

**Affiliations:** 1 Division of Urology, Department of Surgery, Far Eastern Memorial Hospital, New Taipei City 220216, Taiwan; 2 PhD Program in Nutrition & Food Science, Fu Jen Catholic University, New Taipei City 242062, Taiwan; 3 Graduate Institute of Biomedical and Pharmaceutical Science, Fu Jen Catholic University, New Taipei City 242062, Taiwan; 4 Department of Obstetrics and Gynecology, Cathay General Hospital, Taipei 106438, Taiwan; joseph@cgh.org.tw; 5 School of Medicine, Fu Jen Catholic University, New Taipei City 242062, Taiwan; 6 Department of Obstetrics and Gynecology, Taipei City Hospital, Zhongxing Branch and Branch for Women and Children, Taipei 103212, Taiwan; 7 Department of Urology, En Chu Kong Hospital, New Taipei City 237414, Taiwan; 8 Department of Nursing, College of Healthcare & Management, and General Education Center, Asia Eastern University of Science and Technology, New Taipei City 220303, Taiwan; 9 Center for Health Research and Innovation, Fu Jen Catholic University, New Taipei City 242062, Taiwan

**Keywords:** teratozoospermia, CDX4, variant, sperm-head defect

## Abstract

*Background and Objectives*: Male factors account for approximately 30–50% of subfertile couples. Teratozoospermia is one of the major causes of male infertility; however, the genetic factors underlying many cases remain incompletely understood. This study aimed to identify potential genetic variants associated with teratozoospermia and to investigate the possible involvement of Caudal-Type Homeobox 4 (CDX4) in sperm morphogenesis. *Materials and Methods*: Whole-exome sequencing was performed in 44 individuals with teratozoospermia. A rare *CDX4* variant (NM_005193.2, c.103G>T; NP_005184.1, Gly35Cys) was identified and confirmed by Sanger sequencing. Because *CDX4* is located on the X chromosome, this variant was interpreted as hemizygous in the male patient. Sperm morphology, CDX4 localization, public GEO transcriptomic data (GSE6969), and CDX4 expression during murine spermiogenesis were analyzed. *Results*: The CDX4 p.Gly35Cys variant is located within the Caudal-like transactivation domain, a conserved region involved in transcriptional regulation. Spermatozoa from the patient carrying this variant exhibited severe morphological abnormalities, predominantly involving sperm-head defects, together with aberrant CDX4 localization along the midpiece and tail, in contrast to the neck- and annulus-enriched distribution observed in control spermatozoa. Reanalysis of the GEO dataset showed increased CDX4 transcript levels in teratozoospermic samples compared with normozoospermic controls. During murine spermiogenesis, CDX4 was detected in the nuclei of spermatogonia and spermatocytes and subsequently localized to the sperm head and neck/tail regions during sperm morphogenesis. *Conclusions*: *CDX4* c.103G>T (p.Gly35Cys) is a rare hemizygous X-linked candidate variant associated with severe teratozoospermia in a single patient. Further cohort-based, segregation, and functional studies are required to clarify its role in sperm morphogenesis.

## 1. Introduction

### 1.1. Global Burden of Infertility

Infertility is a major global health challenge, affecting 10–15% of couples worldwide [1]. Current epidemiological data estimate that approximately 187 million couples face reproductive difficulties globally. This prevalence establishes infertility as a critical public health issue. Notably, male factors contribute to nearly half of all infertility cases [2,3]. Consequently, male infertility has become a primary focus for research and clinical strategies aimed at improving reproductive outcomes. The etiology of male infertility is highly heterogeneous and includes anatomical abnormalities (e.g., varicocele), endocrine disorders, infections, environmental factors such as oxidative stress, and genetic abnormalities [2,4]. Teratozoospermia is one of the leading clinical manifestations of male sterility, and the etiology of teratozoospermia is heterogeneous, including genetic, environmental, oxidative, endocrine, infectious, and idiopathic factors [5,6].

### 1.2. Whole-Exome Sequencing Enables Male Infertility Gene Discovery

The advent of whole-exome sequencing (WES) technology over the past decade has dramatically accelerated the discovery of novel disease-causing genetic changes (e.g., copy number variation, deletion, insertion, and nucleotide alterations) and candidate genes associated with various male infertility phenotypes [7,8,9,10,11,12]. However, because the etiologies of male infertility are likely many, studies usually focused on a particular subgroups of patients, such as men presenting with globozoospermia [12]. Different subgroups of teratozoospermia have been evaluated through NGS, such as sperm with multiple morphological abnormalities of the flagella [8], and cases of primary ciliary dyskinesia-associated male infertility [9]. Recently, our laboratory identified and functionally characterized pathogenic *AGTPBP1* mutations in 12 Taiwanese teratozoospermic cases through WES [13]. In the present study, we expanded the teratozoospermia cohort to 44 cases and identified a novel hemizygous variant in *Caudal-Type Homeobox 4* (*CDX4*) gene.

### 1.3. The Caudal-Related Homeobox Gene Family: CDX4

CDX4 is a homeodomain transcription factor that belongs to the caudal-related family of homeobox genes [14]. It contains a caudal-like transactivation domain (amino acids 13–170) and a homeodomain (amino acids 175–230). The Cdx gene family was initially identified and characterized in *Drosophila* and is essential for proper posterior development [15,16]. In mice and humans, there are three members of the Cdx family, *Cdx1, Cdx2*, and *Cdx4*, all of which are involved in early anteroposterior patterning and axial elongation through upstream regulation of the *Hox* gene [17]. The functions of CDX1 and CDX2 in intestinal tissue homeostasis and gastrointestinal cancers have been well studied [18]. CDX4 participates in hematopoiesis in humans and mice, and is expressed in patients with acute erythroid leukemia (AEL), a more malignant and uncommon form of acute leukemia. After transplantation of packaging cell lines stably expressing the CDX4 protein, mice also display AEL [19]. Moreover, CDX4 and CDX2 are also critical for placenta development [20]. Despite these well-characterized roles in embryonic development and hematopoietic malignancy, the potential function of CDX4 in human testicular physiology and spermatogenesis has remained completely unexplored and uncharacterized prior to the current investigation.

In this study, we aimed to characterize *CDX4* as a potential genetic contributor identified in a Taiwanese patient with severe teratozoospermia and to explore its possible involvement in sperm formation.

## 2. Materials and Methods

### 2.1. Patient Recruitment and Clinical Evaluation

This study was approved by the Ethics Committee of Cathay General Hospital under the following IRB approval numbers: CGH-FJ105006 (approved on 20 July 2017) and CGH-P102031 (approved on 2 September 2014). Written informed consent was obtained from all participants. All patients underwent a comprehensive clinical evaluation, including assessment of smoking history and physical examination. Semen samples were collected by masturbation after 3–5 days of sexual abstinence. After liquefaction, routine semen parameters (semen volume, sperm concentration, and motility including total and progressive motility) were assessed according to the World Health Organization (WHO) 2010 guidelines, which were the standard clinical protocol used at the time of sample collection and analysis [21]. Sperm morphology was evaluated using Kruger’s strict criteria. Based on the morphology assessment, 44 Taiwanese men with teratozoospermia, characterized by >85% abnormal sperm head morphology among the evaluated spermatozoa, were selected for further systematic genetic screening and comprehensive clinical and laboratory evaluation. Clinical assessments included detailed medical and reproductive histories, a complete physical examination with particular attention to genitourinary anatomy, and standard laboratory testing. Chromosomal abnormalities were excluded by peripheral blood lymphocyte karyotyping. For detailed classification of sperm morphological abnormalities, semen smears were further examined by light microscopy after standard hematoxylin and eosin staining in accordance with established WHO criteria.

### 2.2. Whole-Exome Sequencing and Variant Analysis

Genomic DNA was extracted from sperm using a QIAamp DNA Micro Kit (QIAGEN, Hilden, Germany) according to the manufacturer’s instructions and subjected to whole-exome sequencing (WES) as previously described [13]. Genomic DNA libraries were prepared using the Illumina TruSeq Exome Library Prep Kit (Illumina, San Diego, CA, USA) and sequenced on the Illumina NextSeq platform. The average sequencing depth was approximately 50×. Raw sequencing reads were aligned to the reference genome using the Burrows–Wheeler Aligner (BWA). Raw sequencing reads were aligned to the human reference genome (GRCh37/hg19). Variant calling was then performed using established bioinformatics pipelines to identify single-nucleotide variants (SNVs) and small insertions/deletions (indels). To prioritize candidate pathogenic variants, a stringent multi-step filtering strategy was applied. Variants were initially filtered to exclude common variants (minor allele frequency > 5%) based on public population databases, including the 1000 Genomes Project and gnomAD. Remaining variants were prioritized if they were predicted to be deleterious or have uncertain impact by in silico prediction tools, if they were identified in genes with testis-specific or testis-enriched expression, and if they resided in genes not previously reported or only rarely implicated in male reproductive function. Candidate variants were validated by bidirectional Sanger sequencing of PCR amplicons spanning the variant site. The putative functional impact of CDX4 variants was further evaluated using multiple in silico prediction algorithms, including SIFT, PolyPhen-2, LRT, and MetaSVM. A patient carrying a novel hemizygous missense variant in CDX4 was identified. However, DNA samples from the patient’s mother, father, siblings, and other maternal relatives were not available for segregation analysis.

### 2.3. Immunofluorescence Staining of Spermatozoa

Semen samples from the patient carrying the CDX4 p.Gly35Cys variant and from fertile controls were washed with 1× phosphate-buffered saline (PBS). Immunofluorescence staining was performed as previously described [22], with minor modifications to assess CDX4 localization in human spermatozoa. Spermatozoa were spread onto glass slides, air-dried, and fixed. The slides were permeabilized with 0.1% Triton X-100 and washed twice with Tris-buffered saline (TBS). Samples were then incubated with an anti-CDX4 primary antibody (Affinity Biosciences, Cat. No. DF8285, Cincinnati, OH, USA) diluted in Dako Antibody Diluent (1:500; Agilent Technologies, Santa Clara, CA, USA) for 60 min at room temperature. After primary antibody incubation, the slides were washed twice with TBS for 10 min each with gentle shaking at 100 rpm and then incubated with an Alexa Fluor 488-conjugated goat anti-rabbit secondary antibody. The slides were subsequently washed twice with TBS for 10 min each with gentle shaking at 100 rpm. Nuclei were counterstained with 4′,6-diamidino-2-phenylindole (DAPI; Sigma-Aldrich, St. Louis, MO, USA)), and the sperm midpiece was labeled with MitoTracker Red CMXRos (50 nM) for 10 min. After staining, the slides were washed with TBS for 5 min with gentle shaking at 100 rpm. Finally, the samples were mounted using Dako mounting medium and examined by fluorescence microscopy to assess CDX4 localization.

### 2.4. Histology and Immunofluorescence Staining

All procedures were approved by the institutional animal care and use committee (Approval No: A11068, approved on 29 April 2022). Adult C57BL/6 male mice (8–12 weeks) were housed under standard conditions. Testes from adult mice were collected and fixed in 4% paraformaldehyde (PFA) in PBS at 4 °C overnight. The procedures were performed as previously described [13]. Paraffin-embedded testis sections (3 μm) were deparaffinized in xylene and rehydrated through a graded ethanol series. Briefly, sections were incubated twice in xylene for 10 min each, followed by 100% ethanol twice for 5 min each, 95% ethanol for 5 min, 80% ethanol for 5 min, and 70% ethanol for 5 min, and were finally rinsed in distilled water. Antigen retrieval was then performed by boiling the sections in citrate buffer (pH 6.0) for 10 min. After blocking with 5% normal goat serum for 60 min at room temperature, the sections were incubated overnight at 4 °C with an anti-CDX4 primary antibody diluted in Dako Antibody Diluent (Agilent/Dako; 1:500). The sections were then washed twice with Tris-buffered saline (TBS) for 10 min each. CDX4 localization was detected using an Alexa Fluor 488-conjugated secondary antibody (1:1000), followed by two washes with TBS for 10 min each. Nuclei were counterstained with DAPI, and the acrosome was visualized using peanut agglutinin (PNA; L-32458; Invitrogen, Thermo Fisher Scientific, Waltham, MA, USA). Fluorescence images were acquired using a Leica DM2500 LED fluorescence microscope equipped with a Leica DFC7000 T camera (Leica Microsystems, Wetzlar, Germany)**.** Images were captured with an exposure time of 0.5 s and used to assess CDX4 localization in different germ-cell types and at different stages of the seminiferous epithelium.

### 2.5. Statistical Analysis

Statistical analyses were performed using GraphPad Prism 5 (GraphPad Software, San Diego, CA, USA). For the reanalysis of the public transcriptomic dataset, CDX4 transcript levels were compared between normozoospermic controls (*n* = 12) and teratozoospermic cases (*n* = 7). Data are presented as the mean ± standard deviation (SD), with ranges provided where appropriate. Because of the relatively small sample size and the comparison of two independent groups, differences between groups were analyzed using a two-tailed Mann–Whitney U test. No missing values were included in the analyzed dataset. A value of *p* < 0.05 was considered statistically significant.

## 3. Results

### 3.1. Novel Hemizygous CDX4 p.Gly35Cys Genetic Alteration Identified in a Teratozoospermia Patient

**Figure 1:**
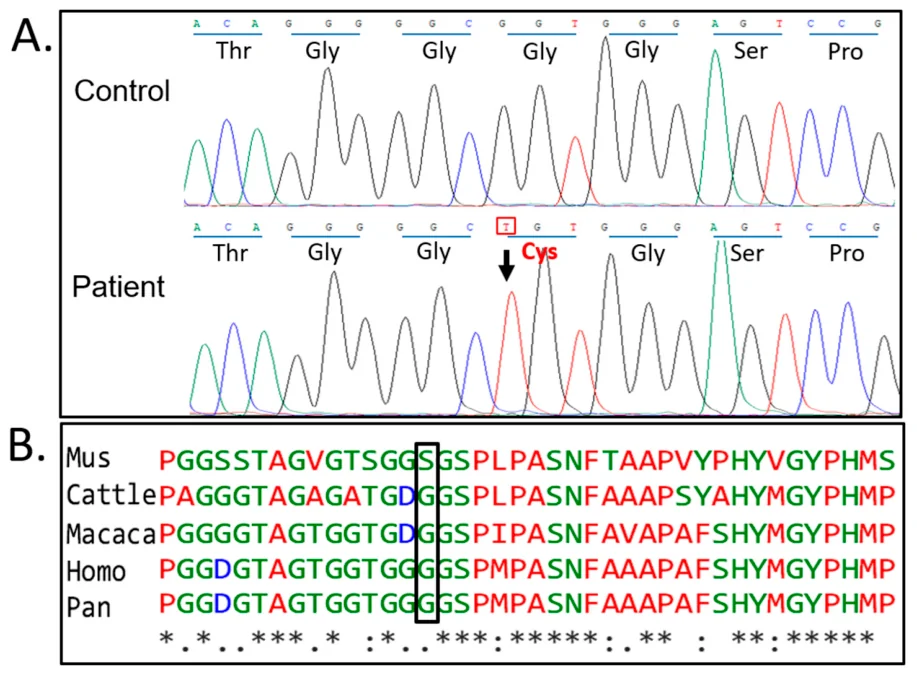
Identification and Evolutionary Conservation of the Hemizygous Novel *CDX4* c.103G>T(p.Gly35Cys) Variant. (**A**) Sanger sequencing chromatograms confirmed the *CDX4* c.103G>T variant in the patient (**bottom**) compared with the fertile control (**top**), showing a clear G-to-T transversion (arrow; red box). (**B**) Multiple sequence alignment of CDX4 orthologs from five mammalian species—*Mus musculus* (Mus), *Bos taurus* (Cattle), *Macaca mulatta* (Macaca), *Homo sapiens* (Homo), and *Pan troglodytes* (Pan)—performed using Clustal Omega demonstrates evolutionary conservation of Gly35, the residue affected by the patient variant. “*” indicates a conserved residue; “.” indicates weak conservation; “:” indicates no conservation.

Through whole-exome sequencing of a 44-patient teratozoospermia cohort, we identified a novel hemizygous missense variant in *CDX4* (NM_005193.2, c.103G>T; NP_005184.1, Gly35Cys). Because *CDX4* is located on the X chromosome, this variant was interpreted as hemizygous in this male patient. The index patient reported regular sexual activity with his spouse and had no history of varicocele, cryptorchidism, infection, or smoking. No chromosomal abnormality was detected. Assisted reproductive outcomes were not available. However, DNA samples from the patient’s mother, father, siblings, and other maternal relatives were unavailable; therefore, segregation analysis could not be performed. Semen analysis demonstrated normal semen volume (3.0 mL), sperm concentration (34 × 10^6^/mL), and total motility (50%), all within WHO reference ranges (Table 1). However, only 3.02% of spermatozoa exhibited normal morphology, consistent with severe teratozoospermia. Morphological evaluation revealed that sperm head defects accounted for the majority of abnormalities (86.04%), whereas neck defects, tail defects, and cytoplasmic droplets represented 4.53%, 3.77%, and 2.64%, respectively (Table 1). The variant was confirmed by Sanger sequencing (Figure 1A, lower panel), while fertile control subjects (upper panel) exhibited only the wild-type sequence. Multiple sequence alignment using the Clustal Omega (version 1.2.4) algorithm demonstrated that the affected residue, Gly35, is highly conserved among mammalian CDX4 orthologs, including *Mus musculus*, *Bos taurus*, *Macaca mulatta*, *Homo sapiens*, and *Pan troglodytes* (Figure 1B). Collectively, these findings indicate that the hemizygous *CDX4* p.Gly35Cys variant is associated with predominant sperm head abnormalities despite preserved sperm count and motility, suggesting that CDX4 may play a critical role in sperm head morphogenesis during late spermiogenesis.

### 3.2. Population Frequency and Functional Prediction Analysis of the CDX4 c.103G>T (p.Gly35Cys) Variant

Comprehensive interrogation of major population genetic databases demonstrated that the *CDX4* c.103G>T (p.Gly35Cys) variant was absent from the 1000 Genomes Project, gnomAD overall population, gnomAD East Asian subcohort, and dbSNP databases, supporting its classification as a novel or extremely rare variant. This finding suggests that *CDX4* c.103G>T (p.Gly35Cys) may represent a rare candidate variant associated with teratozoospermia in this patient (Table 2). Functional prediction analyses showed partially concordant results, with PolyPhen-2 and Meta-SVM predicting deleterious effects, whereas SIFT and LRT classified the variant as tolerated or neutral. Biochemically, the glycine-to-cysteine substitution represents a non-conservative amino acid change that may disrupt protein conformation. Given the structural flexibility provided by glycine and the reactive thiol-containing side chain of cysteine, this substitution may alter CDX4 protein folding, DNA-binding activity, or nuclear localization, thereby potentially contributing to teratozoospermia pathogenesis.

### 3.3. Aberrant Localization of CDX4 in Spermatozoa Carrying the CDX4 c.103G>T (p.Gly35Cys) Variant

**Figure 2:**
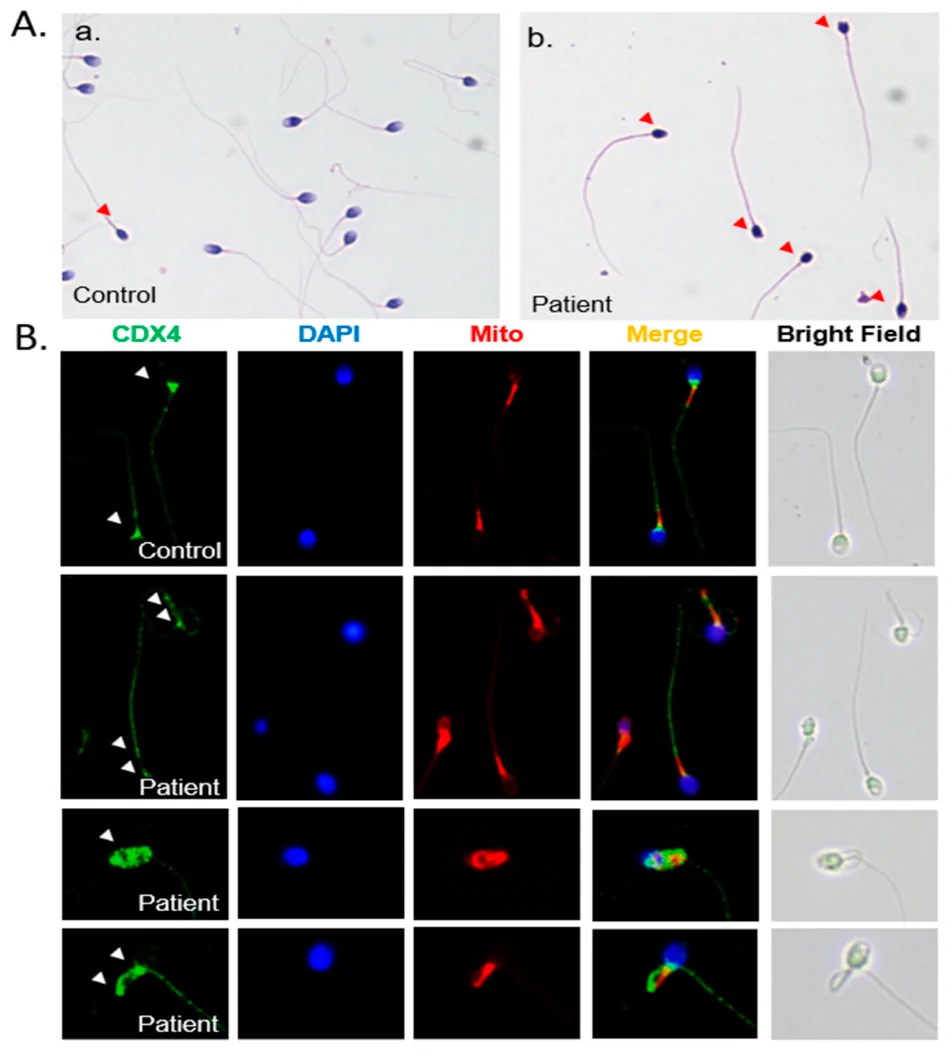
Morphological Defects and Aberrant CDX4 Localization in Spermatozoa from the Patient with the *CDX4* c.103G>T(p.Gly35Cys) Variant. (**A**) Light microscopy of spermatozoa from the patient harboring the hemizygous *CDX4* c.103G>T (p.Gly35Cys) variant (**b**) shows severe teratozoospermia on H&E staining compared with the fertile control (**a**). Red arrows indicate representative abnormalities, including irregularly shaped heads and defective or absent acrosomes. (**B**) Immunofluorescence analysis reveals aberrant CDX4 localization and accumulation in spermatozoa from the patient carrying the *CDX4* c.103G>T variant compared with controls. From left to right: CDX4 (green), nuclei (DAPI; blue), mitochondria (MitoTracker; red), merged image, and bright-field view. White arrows indicate CDX4 signals in spermatozoa from the patient and the controls. Images were acquired at 400× magnification.

To evaluate the morphological characteristics of spermatozoa carrying the *CDX4* c.103G>T (p.Gly35Cys) variant, hematoxylin and eosin (H&E) staining was performed (Figure 2). In fertile control subjects, most spermatozoa exhibited normal oval-shaped heads with uniformly condensed chromatin (Figure 2A(a)). In contrast, spermatozoa from the patient harboring the *CDX4* c.103G>T (p.Gly35Cys) variant displayed abnormal head morphology accompanied by aberrantly condensed chromatin structures (Figure 2A(b), red arrowheads). Furthermore, we performed immunofluorescence staining to assess whether the *CDX4* p.Gly35Cys variant alters the subcellular localization and distribution of CDX4 in human spermatozoa. In fertile control subjects, CDX4 signals were predominantly concentrated at the sperm neck, with only weak staining along the tail (Figure 2B). In contrast, spermatozoa from the patient carrying *CDX4* p.Gly35Cys showed dispersed CDX4 signals across both the neck and tail, accompanied by abnormal sperm morphology (Figure 2B). Notably, spermatozoa with severe head and tail deformities frequently exhibited marked CDX4 accumulation within the sperm head and tail regions (Figure 2B). Collectively, these results suggest that the CDX4 p.Gly35Cys variant disrupts the normal subcellular localization of CDX4 and is closely associated with abnormal sperm morphology.

### 3.4. In Silico Analysis of CDX4 Transcripts in Teratozoospermic Patients

**Figure 3:**
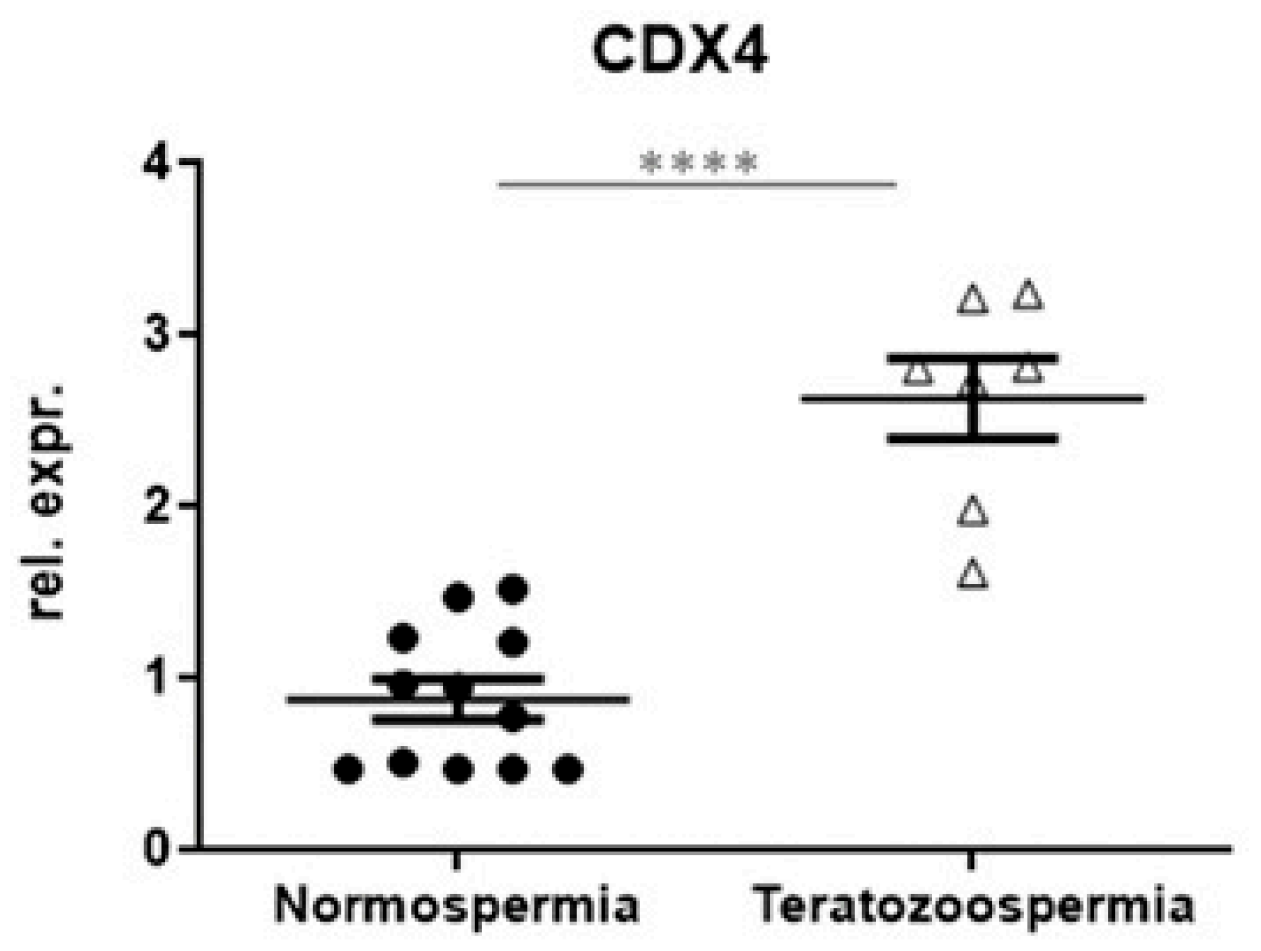
Comparative bioinformatic analysis of CDX4 transcript levels. Reanalysis of human sperm transcriptomic data showed that CDX4 transcript levels were significantly increased in teratozoospermic cases (*n* = 7) compared with normozoospermic controls (*n* = 12), representing an approximately 2.9-fold increase. Data are presented as the mean ± SD. Statistical significance was determined using a two-tailed Mann–Whitney U test (*p* < 0.001). ****p < 0.0001.

To determine whether CDX4 expression is associated with teratozoospermia, we filtered and reanalyzed the data from the cDNA microarray dataset reported by Platts et al. (2007) [23]. To determine whether CDX4 expression is associated with teratozoospermia, we filtered and reanalyzed data from the cDNA microarray dataset reported by Platts et al. (2007) [23]. In this dataset, CDX4 transcript levels in sperm differed markedly between normozoospermic controls (n = 12) and teratozoospermic cases (n = 7), indicating distinct expression profiles between the two groups. Normozoospermic controls showed CDX4 expression levels of 0.91 ± 0.28 relative units (range, 0.42–1.53), with values clustered near baseline. In contrast, teratozoospermic cases exhibited significantly higher CDX4 expression (2.68 ± 0.41 relative units; range, 2.05–3.18), representing an approximately 2.9-fold increase compared with controls (Mann–Whitney U test, *p* < 0.001) (Figure 3). Based on the results presented in Figure 3, we suggest that CDX4 transcript levels may be dysregulated in teratozoospermic samples.

### 3.5. Dynamic Expressional Patterns of CDX4 During Murine Spermatogenesis

**Figure 4:**
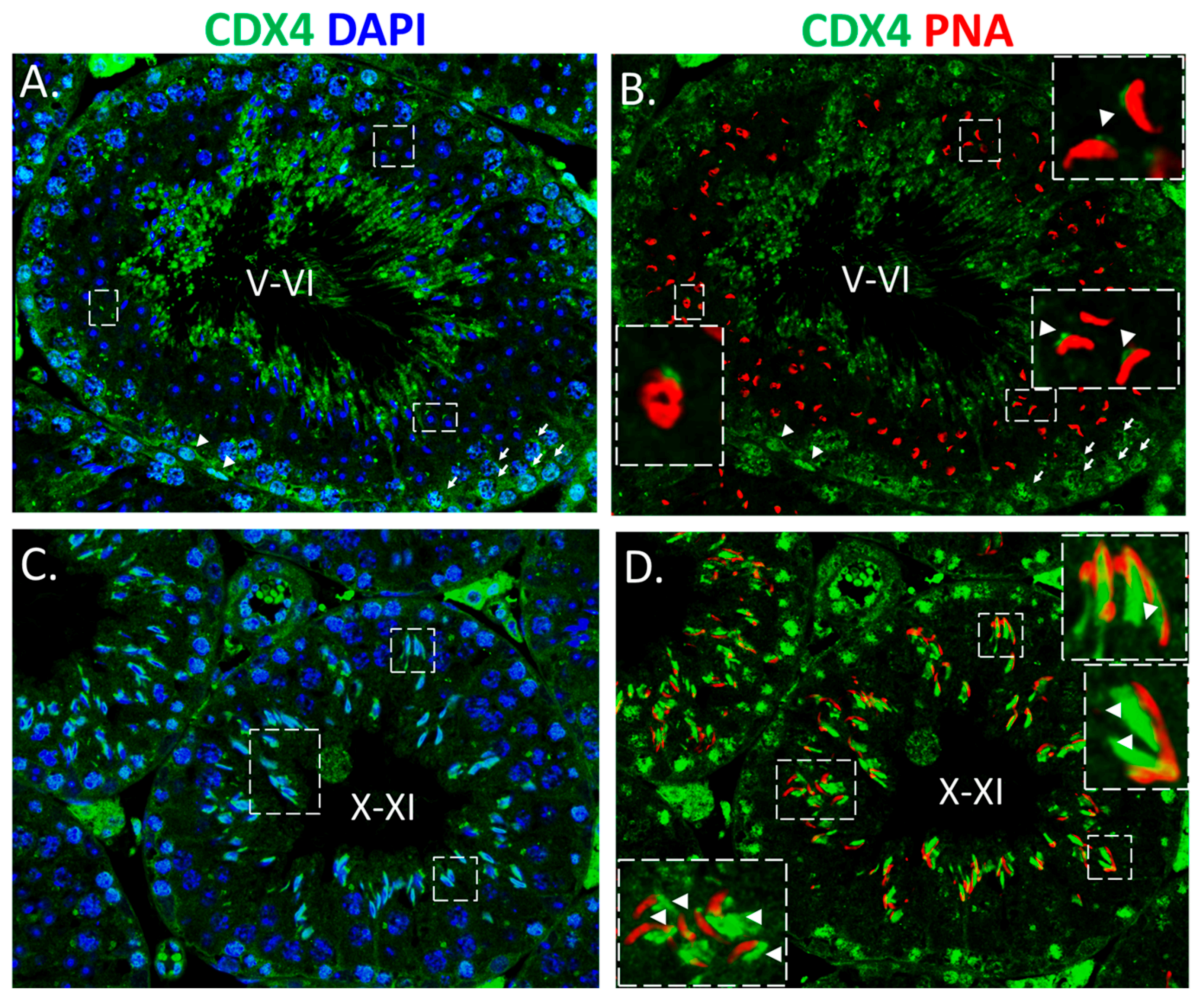
Stage-specific localization of CDX4 during mouse spermatogenesis. Mouse testicular sections were subjected to immunofluorescence staining to examine CDX4 distribution at early (stages V–VI) and late (stages X–XI) spermatogenic waves. CDX4 is shown in green, nuclei are counterstained with DAPI (blue), and the acrosome is labeled with lectin (red). Panels (**A**,**C**) show merged images of CDX4 (green) with DAPI (blue), whereas panels (**B**,**D**) show merged images of CDX4 (green) with lectin (red). At stages V–VI (**A**,**B**), CDX4 signals were weakly nuclear in spermatogonia (arrowheads) and spermatocytes (arrows), and were additionally enriched at the apical region of elongating spermatids (boxed areas; arrowheads). At stages X–XI (**C**,**D**), CDX4 signals became strongly stained at the shaping sperm head in elongating spermatids (boxed areas; arrowheads). Images were acquired at 400× magnification.

**Figure 5:**
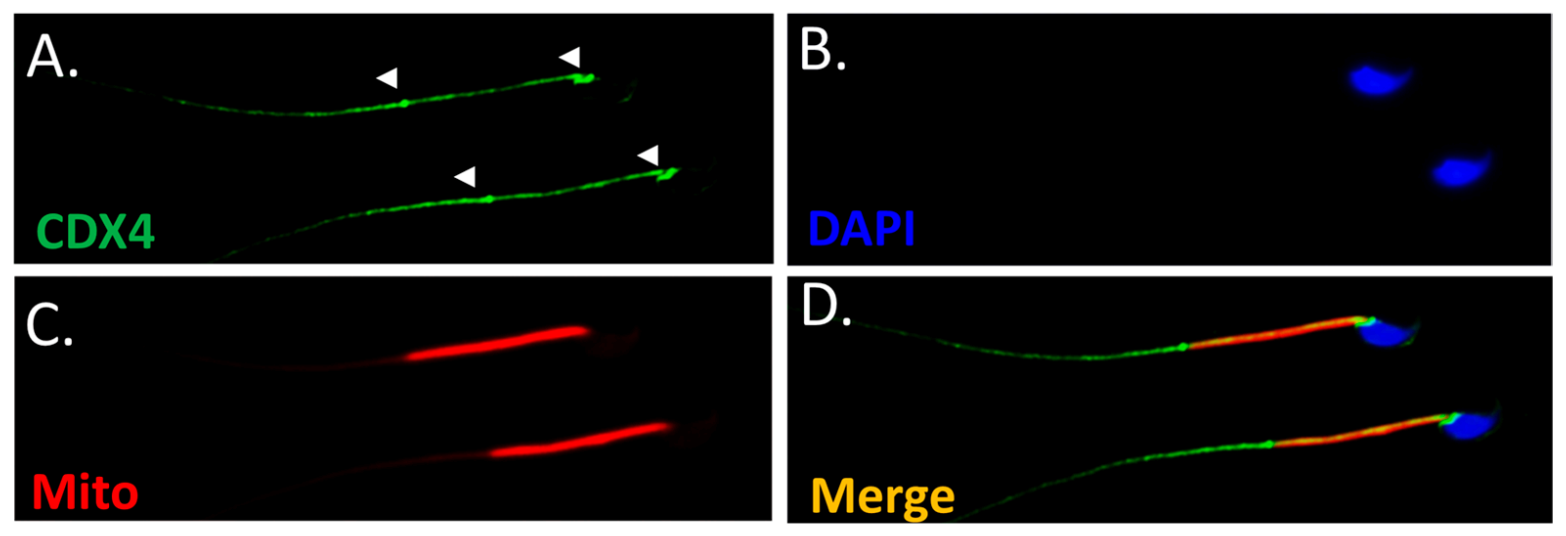
Subcellular localization of CDX4 in mouse spermatozoa. Immunofluorescence staining was performed to visualize CDX4 distribution in mouse spermatozoa. (**A**) CDX4 (green). White arrows indicate the main CDX4 signals. (**B**) Nuclei counterstained with DAPI (blue). (**C**) Mitochondria labeled with MitoTracker (red). (**D**) Merged images of CDX4, DAPI, and mitochondrial signals. Images were acquired at 400× magnification.

Based on the results presented in Figure 1, Figure 2 and Figure 3 and Table 1 and Table 2, genetic alteration and transcriptional dysregulation of CDX4 are strongly associated with sperm morphological defects, particularly involving the sperm head. To further investigate whether CDX4 participates in sperm head shaping and tail elongation, we examined its dynamic, stage-dependent localization during murine spermatogenesis by immunofluorescence staining of testicular sections (Figure 4). At stages V–VI, CDX4 signals were predominantly nuclear, with weaker cytoplasmic staining, in spermatogonia (Figure 4A,B; arrowheads) and spermatocytes (Figure 4A,B; arrows). Notably, at the same stages, CDX4 was enriched at the apical tip of the acrosome in elongating spermatids (Figure 4B; boxed areas). At later stages (X–XI), CDX4 localization shifted to the manchette structure of the shaping sperm head in elongating spermatids, which was covered by the acrosomal marker PNA (red) (Figure 4D). In mature spermatozoa, CDX4 was enriched at the neck region with weaker staining along the entire tail (Figure 5). Overall, CDX4 localized primarily to the nuclei of spermatogonia and spermatocytes, the sperm head of spermatids, and the neck of mature sperm (Figure 4 and Figure 5), providing biological plausibility for the severe sperm head defects observed in teratozoospermic patients carrying the hemizygous CDX4 variant.

## 4. Discussion

In this study, we integrate clinical phenotyping, human genetics, and stage-resolved expression data to implicate CDX4 in sperm morphogenesis and teratozoospermia. Whole-exome sequencing of a 44-patient cohort identified a novel hemizygous *CDX4* missense variant (c.103G>T, p.Gly35Cys) in a patient with severe teratozoospermia dominated by sperm head defects, despite preserved sperm concentration and motility. Immunofluorescence further revealed aberrant CDX4 localization in patient spermatozoa, with dispersed signals extending beyond the typical neck/annulus-enriched pattern in controls and frequent accumulation in morphologically abnormal sperm. To clarify biological plausibility, we examined CDX4 localization during murine spermatogenesis. CDX4 was enriched at the apical tip of the acrosome in early elongating spermatids, then shifted to the manchette-associated region of the shaping sperm head, and ultimately became concentrated at the sperm neck and tail in mature sperm. Together, these findings suggest that CDX4 may be a potential genetic contributor to teratozoospermia and may be involved in abnormal sperm morphology, particularly impaired sperm head shaping during late spermiogenesis.

### 4.1. Our Case Represents Polymorphic Teratozoospermia

Teratozoospermia can be broadly classified into five major subtypes (reviewed by Kleshchev MA et al.) [24,25]: (1) globozoospermia, in which 70–100% of spermatozoa display round heads with an absent acrosome; (2) multiple morphological abnormalities of the sperm flagella (MMAF), characterized by 70–100% of spermatozoa exhibiting multiple combined flagellar defects, including short, coiled, and irregularly thick flagella; (3) dysplasia of the fibrous sheath (DFS), where essentially 100% of spermatozoa show short flagella with irregular thickness; (4) acephalic spermatozoa syndrome, in which 90–100% of spermatozoa have heads detached from the flagella; and (5) polymorphic teratozoospermia, in which 96–100% of spermatozoa exhibit diverse morphological abnormalities, such as amorphous heads, acrosomal defects, and vacuolated heads. In this study, the patient carrying the novel hemizygous *CDX4* p.Gly35Cys variant exhibited severe sperm morphological abnormalities, affecting 96.98% of spermatozoa. Head defects were predominant, accounting for 86.04% of spermatozoa, whereas neck defects, tail defects, and cytoplasmic droplets accounted for 4.53%, 3.77%, and 2.64%, respectively. According to the ACMG/AMP variant interpretation guidelines [26], the *CDX4* c.103G>T (p.Gly35Cys) variant was classified as a variant of uncertain significance (VUS) based on its rarity or absence in public population databases, conservation of the affected residue, and supportive in silico predictions. However, owing to the lack of family segregation analysis and direct functional validation, this variant cannot currently be classified as pathogenic or likely pathogenic. This pattern is consistent with polymorphic teratozoospermia, which is characterized by multiple concurrent sperm morphological abnormalities. Several genes, including *DNAH6*, *IFT140*, *KATNAL2*, *SEPTIN12*, *SPATA16*, *SOHLH1*, and *TAF7L*, have been reported to harbor pathogenic or likely pathogenic variants associated with this phenotype [6,24,27]. In our study, CDX4 localization most closely aligns with the head-shaping/acrosome–manchette module, as it localizes to the apical acrosomal tip in early elongating spermatids and later shifts to the manchette-associated region of the shaping sperm head, supporting a mechanistic link to the predominant sperm-head defects observed in the patient carrying hemizygous *CDX4* p.Gly35Cys variant.

### 4.2. Genetic Variants of Transcription Factor-Related Genes in Teratozoospermia Cases

Pathogenic variants causing polymorphic teratozoospermia in humans have been reported in genes whose products can be functionally classified into several categories: (1) assembly and function of the acroplaxome and manchette, (2) intracellular transport, (3) cytoskeletal organization/function, (4) transcription factors and transcriptional regulators, (5) ubiquitination and autophagy, and (6) others [25,27]. CDX4 is a homeodomain transcription factor containing a caudal-like transactivation domain and a homeodomain. Accordingly, CDX4 can be placed in the “transcription factors and transcriptional regulators” category, which includes genes such as *SOHLH1*, *TAF7L*, *CEP112*, *KCTD19*, *ZMYND15*, *TENT5D*, and *NANOS1*. For example, SOHLH1 is a germ cell–specific transcription factor and a key regulator of oocyte and spermatogonial maturation and differentiation through binding and transactivation of the *Lhx8*, *Zp1*, and *Zp3* promoters [28]. A pathogenic splice-site variant in *SOHLH1* (c.346-1G>A) has been reported in patients with non-obstructive azoospermia [29] and polymorphic teratozoospermia [30]. Previous studies have shown that CDX4 primarily functions within the HOX regulatory network. CDX4 directly activates HOXA10 transcription by binding to a cis-regulatory element in the HOXA10 promoter, while HOXA10 can in turn activate CDX4, forming a positive feedback loop in myeloid cells [14]. In addition, CDX4 cooperates with Menin to activate *HOXA9* expression in hematopoietic progenitors, and Cdx4 deficiency has been associated with dysregulated expression of multiple posterior Hox genes, including hoxb4, hoxb5a, hoxb6b, hoxb7a, hoxb8a, hoxb8b, and hoxa9a [31]. However, the genes directly regulated by CDX4 during spermatogenesis remain completely unknown. Given that homeobox family genes play critical roles in mammalian spermatogenesis [32,33], it is possible that CDX4 may regulate a subset of developmental or morphogenesis-related homeobox genes involved in sperm formation.

### 4.3. Identification of Genetic Alteration from Taiwanese Teratozoospermia Cases

The genetic investigation of teratozoospermia has become one of the most productive areas in male infertility research, largely driven by next-generation sequencing and Sanger sequencing. Because of substantial genetic heterogeneity, disease-associated variants often differ among populations. In Taiwan, mutations in *SEPT12*, *SEPT14*, and *AGTPBP1* have been identified as teratozoospermia-associated genes [13,34,35]. Two missense mutations in *SEPT12* (c.266C>T; p.Thr89Met and c.589G>A; p.Asp197Asn) and one splice-disrupting transition (c.474G>A) have been reported in association with teratozoospermia. In *SEPT14,* two novel missense mutations, c.367G>A (p.Ala123Thr) and c.998T>C (p.Ile333Thr), have been identified. In addition, three *AGTPBP1* variants have been reported: c.1336A>T (p.Glu423Asp), c.1959C>T (p.Pro653Leu), and c.2499G>A (p.Arg811His). Further analyses using genetically modified murine models have also demonstrated that loss of these genes causes sperm morphological defects, with phenotypes resembling those observed in clinical cases of teratozoospermia [13,22,36]. In this study, we identified a novel hemizygous *CDX4* missense variant (c.103G>T, p.Gly35Cys) in a 44-patient teratozoospermia cohort. Although the patient showed normal sperm concentration and motility, 96.98% of spermatozoa exhibited severe morphological abnormalities, predominantly head defects (86.04%), with fewer neck defects, tail defects, and cytoplasmic droplets. Genetic database screening and functional prediction analyses indicated that this variant is novel and potentially deleterious; the glycine-to-cysteine substitution may alter CDX4 protein conformation, DNA-binding activity, or nuclear localization, thereby contributing to teratozoospermia pathogenesis.

### 4.4. Limitations and Interpretation of the Findings

This study has several limitations. First, only one patient carrying the *CDX4* c.103G>T (p.Gly35Cys) variant was identified in this cohort, and DNA samples from the patient’s mother and other family members were not available for segregation analysis. Second, direct functional validation of this variant was not performed. Third, the immunofluorescence findings were descriptive and were not quantitatively analyzed. Fourth, the GEO analysis should be interpreted only as supportive evidence suggesting possible dysregulation of CDX4 transcript levels in teratozoospermic samples. The GSE6969 dataset was derived from a different population and does not contain information on the specific CDX4 p.Gly35Cys variant identified in our patient. In addition, differences in sperm transcript levels may reflect altered sperm morphology, altered RNA retention, differences in germ-cell composition, or possible contamination by immature germ cells or leukocytes, rather than direct CDX4-driven pathology. Therefore, this dataset cannot validate the pathogenicity of this specific variant. Fifth, CDX4 localization during spermatogenesis was examined in murine testicular tissues, and extrapolation of these findings to human spermatogenesis should therefore be interpreted cautiously. Overall, our results suggest only that CDX4 alteration may be associated with teratozoospermia and may represent a potential genetic contributor. Larger cohorts, family-based segregation analysis, and functional studies are required to establish causality.

## 5. Conclusions

In conclusion, we identified a novel hemizygous *CDX4* missense variant (c.103G>T, p.Gly35Cys) in a Taiwanese patient with severe polymorphic teratozoospermia characterized predominantly by sperm-head defects. Although sperm concentration and motility were preserved, the patient showed marked sperm morphological abnormalities and aberrant CDX4 localization in spermatozoa. Stage-specific analysis in mouse testes further showed dynamic CDX4 localization at the apical acrosomal region, manchette-associated shaping of the sperm head, and mature sperm neck, supporting its potential involvement in sperm head morphogenesis. Together, these findings suggest that CDX4 alteration and dysregulation may contribute to teratozoospermia pathogenesis, although further functional studies and larger cohorts are required to confirm its causality.

## Figures and Tables

**Table 1:** Clinical Features and Semen Analysis of Patient with *CDX4* p.G35C.

Clinical Features	Patient Value	Normal Reference	Status
Age (years)	30	—	—
Karyotype	46, XY	46, XY	Normal
Genetic alteration site	CDX4 c.103G>T p. Gly35Cys	NM_005193.2 NP_005184.1	Hemizygous
**Semen Parameter**		**Normal Range**	**Status**
Volume (mL)	3.0	≥1.5 mL	Normal
Sperm Concentration (×10^6^/mL)	34	>15 × 10^6^/mL	Normal
Sperm Motility	50%	≥40%	Normal
Sperm Morphology	3.02%	≥4%	Abnormal

**Table 2:** Allele Frequency and In Silico Functional Prediction of the CDX4.

c.103G>T (p.Gly35Cys) Genetic Change	
Allele frequency in population *	**Status**
1000 Genomes Project	Not found
All Individuals in gnomAD	Not found
East Asians in gnomAD	Not found
dbSNP	Not found
Functional prediction *	**Status**
Sorting Intolerant From Tolerant (SIFT)	Tolerated (T)
Polymorphism Phenotyping v2 (PolyPhen-2)	Probably Damaging (PD)
Likelihood Ratio Test (LRT)	Neutral (N)
Meta-Support Vector Machine (Meta-SVM)	Damaging (D)

* Accessed on 4 August 2022.

## Data Availability

The original contributions presented in this study are included in the article. Further inquiries can be directed to the corresponding authors.
